# Arginine to glutamine mutation in the substrate binding region impaired the isopentenyl activity of *Mycobacterium tuberculosis *MiaA

**DOI:** 10.22099/mbrc.2023.47247.1825

**Published:** 2024

**Authors:** Smitha Soman, Siya Ram

**Affiliations:** 1 School of Biotechnology, Gautam Buddha University, Gautam Budh Nagar, Greater Noida, Uttar Pradesh, India; 2 School of Sciences, Indira Gandhi National Open University, Maidan Garhi, New Delhi, India

**Keywords:** MiaAn DMAPPn SDMn M. tuberculosisn i6An isopentenylation

## Abstract

tRNAs act as adaptors during protein synthesis and are chemically modified post-transcriptionally for their structural stability as well as accuracy of the translation. Hypomodifications of tRNAs are known to cause various human diseases, including cancer. Studies in bacteria and yeasts showed that levels of tRNA modifications vary under different stress conditions, enabling the organism to modulate gene expression for survival. Isopentelylation of the base 37 (i^6^A37) in the anticodon stem-loop by tRNA isopentenyltransferase (MiaA) is well-conserved modification present in prokaryotes and eukaryotes. i^6^A37 modification increases both the speed and fidelity of translation. A homozygous p.Arg323Gln mutation in the tRNA binding region of tRNA isopentenyltransferase reduced i^6^A37 levels in humans, affecting mitochondrial translation and thereby causing neurodevelopmental disorder. In this study, we mutated the Arg residue at the conserved position to Gln in *Mycobacterium tuberculosis* (M. tb) MiaA and analyzed the i6A modification activity of the enzyme on its target tRNAs. We found that p.Arg274Gln mutant MiaA could not modify the target tRNAs, tRNA^Leu^CAA, tRNA^Phe^GAA, and tRNA^Ser^CGA from M. tb, confirming the role of Arg residue in tRNA binding.

## INTRODUCTION

tRNAs are chemically modified post-transcriptionally to ensure stability and translational fidelity. While many modifications in the tRNA core help maintain the structural integrity and prevent degradation of the tRNAs, modifications in the anticodon stem loop (ASL) play a crucial role in codon: anticodon interaction and decoding. Among these, modifications at position 37 are vital to prevent translational frameshifting [1]. One of the oldest known base modifications at position 37 is N6-isopentenyl adenosine (i^6^A), which is present in all organisms, from eubacteria to mammals. This modification prevents illicit hydrogen bond formation between U33 and A37. It stabilizes Watson-Crick base pairing between A-U base pairs on the first codon position (base 36 of tRNA) by base stacking. Thus the presence of the i^6^A group at position 37 stabilizes the anticodon loop and prevents frameshifting during translation [2]. The target tRNAs for the i^6^A modification enzyme, MiaA in bacteria and TRIT1 in humans, possess a conserved A36-A37-A38 recognition sequence, and dimethylallyl pyrophosphate (DMAPP) provides the isopentenyl group [3]. tRNA isopentenyl transferase gene has homologs in all organisms, though the eukaryotic enzyme has a zinc finger domain which is absent in bacteria [4,5].

Hypomodifications in tRNAs can affect the efficiency of protein synthesis and lead to several human disorders, such as neurological disorders, mitochondrial diseases, and cancer. A homozygous p.Arg323Gln mutation in the ASL binding region of TRIT1 is associated with a neurodevelopmental disease. Patients with this condition have multiple defects in oxidative phosphorylation in skeletal muscles due to deficient mitochondrial tRNA modification. Arg323 amino acid is essential for substrate binding and is conserved in TRIT1 protein and its orthologs in eukaryotes, excluding *S. pombe* *and S. cerevisiae. *In yeast and many bacteria, including *E. coli*, lysine replaces arginine, which has similar properties [6]. 

We have previously identified tRNA^Leu^CAA, tRNA^Phe^GAA, tRNA^Trp^CCA, and tRNA^Ser^CGA tRNAs as targets of MiaA in *Mycobacterium tuberculosis *(M. tb) [7]. To check whether Arg to Gln mutation affects the catalytic activity of M. tb MiaA, we made a site-directed mutation in that location (R274Q), followed by an isopentenylation assay of the target tRNAs by the mutant MiaA. We also made an R274K mutant version of MiaA, which resembles other bacteria, to see how the mutant enzyme acts on target tRNAs.

## MATERIALS AND METHODS

**Bacterial strain and culture conditions:** *Mycobacterium smegmatis* mc^2^155 was used to express and purify M. tb MiaA mutant proteins. The bacterial cells were cultured in the broth of Middlebrook 7H9, in addition to 10% ADC (albumin, dextrose, and catalase) and 0.1% Tween-80. *M. smegmatis* transformed with recombinant *miaA *plasmids were grown on 7H10 agar supplemented with 10% OADC in the presence of Kanamycin (20 µg/ml). 1M stock of Isovaleronitrile (IVN) for protein induction was prepared in DMF and added to 7H9 broth during bacterial inoculation [7].

**Generation of plasmid constructs: **To clone *miaA* in *E.coli *DH5, Rv2727c was PCR amplified from M. tb strain H37Rv. Gene-specific primers were designed with restriction sites *Not*I and *Nde*I and PCR amplified using Phusion DNA polymerase (Thermo Fisher Scientific). The amplicon was digested with *Not*I-*Nde*I and cloned into the corresponding site on pET28(a). 

**Site directed mutagenesis of MiaA:** Site directed mutations (SDM) were done at 274 position of MiaA, changing the amino acid Arginine to Glutamine (R274Q) and Lysin (R274K). pET28(a)-*miaA* was used as a template for SDM. Primers were designed with desired mutations (MiaA R274Q Forward: 5'acccgccgctacgtgcagcggcagcggtcctgg3' and Reverse: 5' ccaggaccgctg ccgctgcacgtagcggcgggt3'; MiaA R274K Forward: 5' acccgccgctacgtgaagcggcagcggtcctgg3' and Reverse: 5' ccaggaccgctgccgcttcacgtagcggcgggt 3') and PCR amplification was carried out for 12 cycles at 95^o^C for 50 sec, 60^o^C for 50 sec & 68^o^C for 18 min, followed by 7 minutes extension at 68^o^C. After PCR, the products were digested with 1ul (20U) of *Dpn*I at 37^o^C for 2hr. Two controls were also done, one without DNA polymerase but with *Dpn*I and other without both DNA polymerase and *Dpn*I. All digested products (Controls & Test) were transformed into *E.coli* DH5α and plated on LB media with kanamycin. Plasmids were isolated and sequenced to confirm the mutations.

**Generation of expression vectors pNit3xFLAG-miaA R274Q & R274K:** Mutant *miaA* inserts were released from pET28-miaA R274Q & pET28-miaA R274K plasmids and re-cloned into *Nde*I-*Hind*III site of pNit3xF-*miaA*. Plasmids were electroporated into electrocompetent *M. smegmatis* at 25kV, 25mF, with the pulse controller resistance of 1000W in an electroporation chamber. The cells were allowed to recover for three hours in 7H9 broth without antibiotics and plated on 7H10 agar with kanamycin for selection [8].

**M. tb MiaA expression in** ***Mycobacterium smegmatis*** **:** *M. smegmatis* transformed with pNit3XF-miaAR274Q and pNit3XF-miaAR274K were inoculated in 7H9 broth with kanamycin at 0.025 OD. 5μM IVN was added to the media prior to inoculation for protein induction. *M. smegmatis *pNit3XF-miaA was included as a control. Immunoprecipitation of mutant proteins was done from 1mg whole cell lysate of *M. smegmatis* using FLAG-M2 affinity gel beads. Western blotting was used to confirm MiaA mutant proteins (38kDa) and protein estimation was performed as previously described [7].

***In vitro*** **transcription of M. tb i6A target tRNAs and isopentenyl transferase assay:** The genes encoding MiaA target tRNAs, tRNA^Leu^CAA (*leuV*), tRNA^Phe^GAA (*pheU*), tRNA^Trp^CCA (*trpT*), and tRNA^Ser^CGA (*serX*), were PCR amplified from M. tb H37Rv DNA. Gene-specific primers were designed with a T7 promoter sequence at the 5' end (5'TAA TACGACTCACTATAGG3') of each forward primer. These tRNAs were transcribed in vitro for 3 hours using PCR Product, T7 RNA Polymerase, dNTP mix (5mM of ATP, GTP, CTP, & UTP), and 32P GTP [7]. The isopentenyl transferase assay was performed using mutant proteins MiaAR274Q and R274K by incubating each target tRNA for 1 hour at 37^o^C in the presence or absence of the substrate dimethylallyl pyrophosphate (DMAPP) (Sigma) [9].

## RESULTS

**Figure 1:**
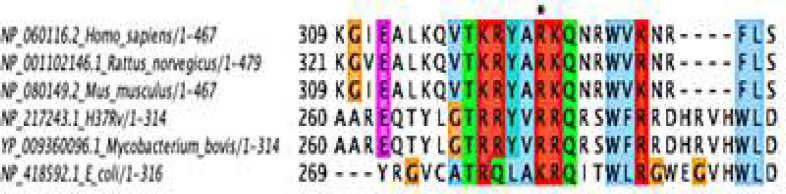
Clustal omega alignment of M. tb MiaA and known orthologs showing the conserved region. Asterisks (*) indicate the position of mutation (R to Q). Mycobacteria has arginine like higher eukaryotes while yeast and other bacteria has lysine

Sequence analysis showed that M. tb MiaA has Arg in its tRNA binding region like eukaryotic isopentenyl transferase (Fig. 1).We generated two mutant versions of MiaA to study the tRNA binding property of the enzyme. Site-directed mutagenesis (SDM) was done at position 274 of MiaA, changing the amino acid Arginine to Glutamine (R274Q) and Lysin (R274K). pET28-miaA was used as the template for SDM. For MiaA R274Q, primers were designed to replace CGA (Arg) with CAG (Glu), and for MiaAR274K, CGA(Arg) was replaced by AAG (K). Plasmids were isolated and sequenced to confirm the mutations. 

**Figure 2:**
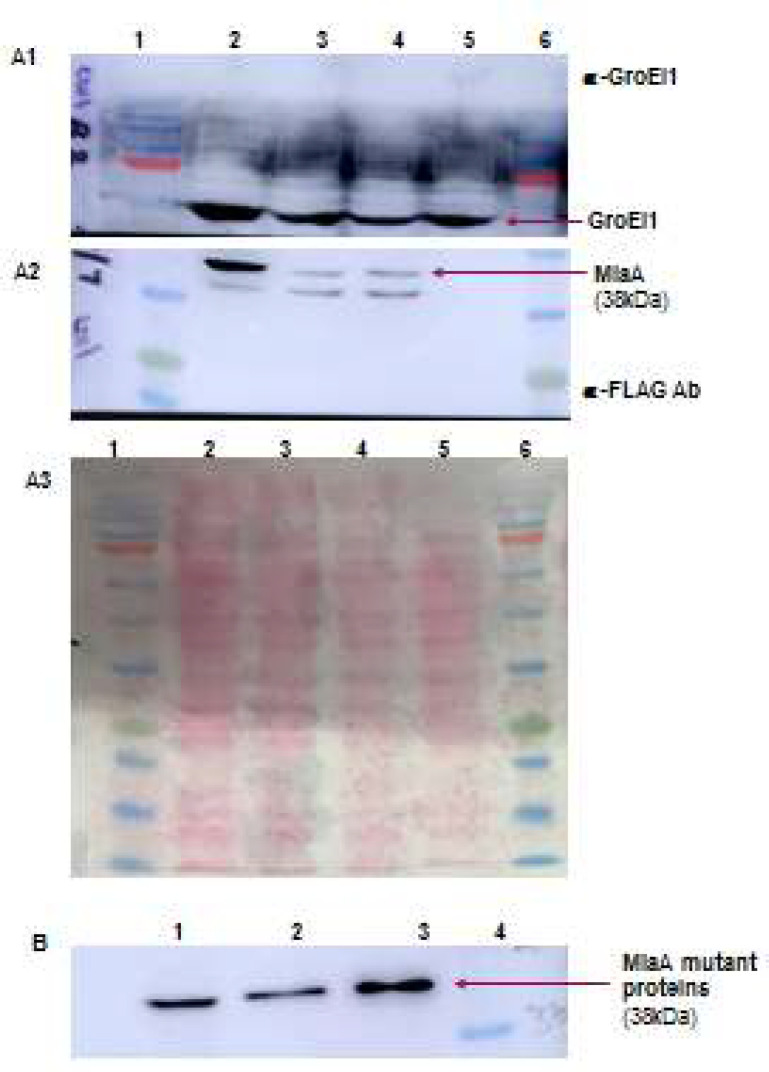
Purification and Immunoprecipitation of MiaA R274Q & R274K. A:Western blot showing purified MiaA mutant proteins probed with FLAG Ab & GroEl1 Ab. Lane1,6- Prestained protein marker, 2-MiaA R274K, 3-MiaA R274Q, 4- MiaA, 5- MiaA uninduced B: Immunoprecipitated proteins. Lane1: MiaA, 2- MiaAR274Q, 3-MiaAR274K, 4-Marker

Mutant miaA genes were cloned into pNit3xFLAG and electroporated into *M. smegmatis*. The expression of the FLAG-tagged MiaA mutant proteins was confirmed by Western blotting with α-FLAG M2 antibody; an antibody against mycobacterial protein GroEL1 was used as the loading control. Bands corresponding to the size of MiaA(38kDa), were obtained for both MiaA R274Q and MiaA R274K (Fig. 2A). Conditions for protein purification and immune-precipitation of mutant proteins were identical to that of MiaA [7]. Protein concentration was found to be 1.28 mg/ml for MiaA R274Q and 1.7mg/ml for R274K (Fig. 2B), as estimated by the BCA protein estimation kit (Thermo Scientific). 

We have previously identified tRNA^Leu^CAA, tRNA^Phe^GAA, tRNA^Trp^CCA, tRNA^Ser^CGA as the tRNA targets of MiaA in *M. tuberculosis* based on the A37A38A39 base sequence and isopentenlyation assay. These tRNAs were synthesized by *in vitro* transcription as described earlier [7]. Target tRNAs were incubated with 5.3mM MiaA R274Q, or R274K along with the substrate DMAPP (0.2mM) and were precipitated after one hour of modification reaction. In each case, the tRNA without the substrate DMAPP served as control. The precipitated tRNAs were digested with RNase T1and separated on 20% polyacrylamide denaturing gel. RNase T1 cleaves guanosines at 3’ end; comparison of tRNA fragments in the presence and absence DMAPP enables the identification of fragments with i6A modification due to mobility shift. The target fragment with i6A modification in tRNA^Leu^CAA and tRNA^Trp^CCA were 12 nucleotides, tRNA^Phe^GAA was 5 nucleotides, and tRNA^Ser^CGA 4 nucleotides. 

**Figure 3:**
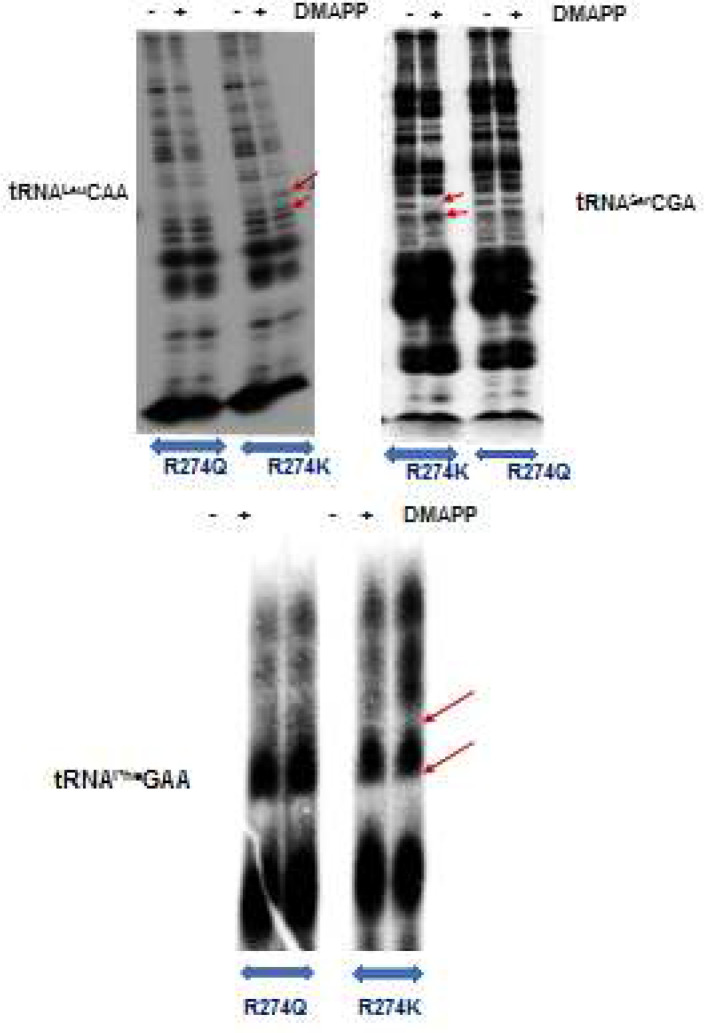
i6A Assay using MiaA mutant proteins R274Q & R274K. Target tRNAs tRNA^Leu^CAA, tRNA^Ser^CGA and tRNA^Ph^eGAA were incubated in the presence or absence of DMAPP. tRNA fragments showed a shift in mobility when incubated with MiaA R274K but no change in band patterns were observed in the presence of MiaA R274Q. The arrows indicate the fragments shifted during migration due to the presence of isopentenyl group

In tRNA^Leu^CAA, tRNA^Phe^GAA, and tRNA^Ser^CGA, isopentenyl assay with MiaA R274Q (Arg to Gln) did not cause any mobility shift among tRNA fragments suggesting the reaction is not taking place. Arginine to Lysine mutation didn't affect the catalytic activity of MiaA as target tRNAs incubated with MiaA R274K showed mobility shift in the fragments with modified residue (Fig: 3). This indicates the presence of Arg at position 274 is essential for M. tb MiaA to bind to its tRNA targets, just like human TRIT1. tRNA fragments were diffused in the case of tRNA^Trp^CCA, making it difficult to distinguish band migration patterns.

## Discussion

i^6^A modification happens when the isopentenyl transferase recognizes the sequence A36-A37-A38 in the substrate tRNA. Structural studies of MiaA from *Pseudomonas aeruginosa* revealed that the DMAPP binding site is formed when the positive charged amino acids on the surface of the enzyme interact with the tRNA[3]. During the binding of the enzyme to the anticodon stem-loop of the tRNA, the base A37 flips out. This flipping of the base causes a conformation change in the MiaA binding pocket, which then allows the substrate binding [4].Changing positive charged arginine to charge-neutral glutamine on the tRNA binding region of MiaA can inhibit the enzyme's catalytic activity, resulting in hypomodification of the tRNA. Eukaryotic isopentenyl transferase has an additional zinc finger in the C terminus, which interacts with nucleotides at the top of the anticodon stem and part of the D loop [5]. The inactivation of *M. tuberculosis* MiaA by Arg to Gln mutation in the tRNA substrate binding site indicates that both mycobacterial and human enzymes interact with the substrate similarly. 

The i6A modified base was one of the first identified tRNA modifications encoded by homologous genes in all organisms, from prokaryotes to humans. In *E.coli*, this modification is needed when the organism moves from one physiological state to another. Like other tRNA modifications, i6A modification regulates gene expression in a codon biased manner. mRNAs enriched with codons sensitive to i6A modifications show biased translation when *E. coli* moves to stationary growth phase [10]. Recent studies revealed that i6A modification is required for fitness and virulence in pathogenic bacteria. In *Shigella flexneri*, *miaA *mutants showed reduced expression of virulence genes, though there were no differences in the mRNA levels of these genes in the mutant strains [11]. *miaA* deletion mutants of Extraintestinal Pathogenic *E. coli* (ExPEC), a major cause of urinary tract infection (UTI) in humans, were unable to colonize the urinary bladder of the host. They also had reduced metabolic flexibility and response against host-induced stresses [12]. Since absence of MiaA can adversely affect the fitness of pathogenic bacteria, *miaA* can be a potential drug target in these organisms.

*Mycobacterium tuberculosis*, the causative organism of tuberculosis, is an intracellular pathogen infecting more than one-third of the global population [13]. Identifying essential genes and unique pathways in this organism will help develop new drugs and vaccines against this deadly pathogen. Though i6A modification is also present in the host (human), the pathway through which the bacteria derive the substrate (2C-methyl-D-erythritol 4-phosphate or MEP pathway) is different from the mevalonate pathway used by eukaryotes. This makes the MEP pathway a unique and promising target for developing new drugs against M. tb [14]. A detailed study of M. tb MiaA will help us to identify the role of this particular modification in the survival and pathogenicity of this bacteria.

## Conflict of Interest:

The authors declare no conflict of interest.

## Authors’ contribution:

SS designed and conducted the experiments and wrote the manuscript. SR conceived of the study and helped in drafting the manuscript. The authors read and approved the final manuscript.
